# Modelling heterogeneity variances in multiple treatment comparison meta-analysis – Are informative priors the better solution?

**DOI:** 10.1186/1471-2288-13-2

**Published:** 2013-01-11

**Authors:** Kristian Thorlund, Lehana Thabane, Edward J Mills

**Affiliations:** 1 Department of Clinical Epidemiology and Biostatistics, McMaster University, Hamilton, ON, Canada; 2 Biostatistics Unit, Father Sean O’Sullivan Research Centre, St Joseph’s Healthcare, Hamilton, ON, Canada; 3 Faculty of Health Sciences, University of Ottawa, Ottawa, ON, Canada

## Abstract

**Background:**

Multiple treatment comparison (MTC) meta-analyses are commonly modeled in a Bayesian framework, and weakly informative priors are typically preferred to mirror familiar data driven frequentist approaches. Random-effects MTCs have commonly modeled heterogeneity under the assumption that the between-trial variance for all involved treatment comparisons are equal (i.e., the ‘common variance’ assumption). This approach ‘borrows strength’ for heterogeneity estimation across treatment comparisons, and thus, ads valuable precision when data is sparse. The homogeneous variance assumption, however, is unrealistic and can severely bias variance estimates. Consequently 95% credible intervals may not retain nominal coverage, and treatment rank probabilities may become distorted. Relaxing the homogeneous variance assumption may be equally problematic due to reduced precision. To regain good precision, moderately informative variance priors or additional mathematical assumptions may be necessary.

**Methods:**

In this paper we describe four novel approaches to modeling heterogeneity variance - two novel model structures, and two approaches for use of moderately informative variance priors. We examine the relative performance of all approaches in two illustrative MTC data sets. We particularly compare between-study heterogeneity estimates and model fits, treatment effect estimates and 95% credible intervals, and treatment rank probabilities.

**Results:**

In both data sets, use of moderately informative variance priors constructed from the pair wise meta-analysis data yielded the best model fit and narrower credible intervals. Imposing consistency equations on variance estimates, assuming variances to be exchangeable, or using empirically informed variance priors also yielded good model fits and narrow credible intervals. The homogeneous variance model yielded high precision at all times, but overall inadequate estimates of between-trial variances. Lastly, treatment rankings were similar among the novel approaches, but considerably different when compared with the homogenous variance approach.

**Conclusions:**

MTC models using a homogenous variance structure appear to perform sub-optimally when between-trial variances vary between comparisons. Using informative variance priors, assuming exchangeability or imposing consistency between heterogeneity variances can all ensure sufficiently reliable and realistic heterogeneity estimation, and thus more reliable MTC inferences. All four approaches should be viable candidates for replacing or supplementing the conventional homogeneous variance MTC model, which is currently the most widely used in practice.

## Background

Multiple treatment comparison (MTC) meta-analysis is an extension of conventional pair wise meta-analysis where only two interventions are being compared at the time. In contrast to pair wise meta-analysis, MTCs allow for simultaneous inferences about the comparative effectiveness and safety of multiple (3 or more) interventions. The statistical models used to analyze meta-analytic data on multiple interventions are commonly employed in the Bayesian frameworks [1] and conventionally employ *non-informative* or *weakly informative* priors for all model parameters (e.g., treatment effects and heterogeneity variances). Such priors are preferred for two main reasons. First, readers are typically already familiar with the purely data driven frequentist approach for pair wise meta-analysis, and use of non-informative or weakly informative priors allows the analysis to, at least theoretically, remain data driven. Second, there is an unfortunate but prevailing concern about use informative priors because such are believed to drive results in the direction of the researchers’ personal believe. While use of informative priors elicited for treatment effect parameters may be inappropriate, it is a misconception that informative priors are necessarily inappropriate for other parameters. This is especially true for parameters where the immediate effect of the informative priors on the treatment effects is not apparent.

Variance parameter estimates play an important role in the overall inferences of an MTC since they impact the width of 95% credible intervals and treatment rank probabilities. A largely under-recognized issue in random-effects MTCs (as well as Bayesian pair wise random-effects meta-analysis) is that apparently weakly informative heterogeneity variance priors may often be moderately informative [2-4], and thus, bias overall inferences to a considerably larger degree than a well thought out informative variance prior would [4-6]. This is particularly relevant in random-effects MTCs where the results of an analysis can change dramatically depending on several factors including number of studies, the amount of heterogeneity between studies [4,7-9].

Another under-recognized issue in random-effects MTCs is the importance of the assumptions made about the similarity and correlation between the degrees of heterogeneity across treatment comparisons (i.e., assumed heterogeneity variance structures) [4,10,11]. Random-effects MTCs have commonly been carried out under the assumption that the between-trial variances representing each of the treatment comparisons are equal (this assumption is also known as the ‘common variance’ or ‘homogeneous variance’ assumption) [12-14]. This approach ‘borrows strength’ for heterogeneity estimation across treatment comparisons, and so, the risk that a weakly informative variance prior unintentionally becomes moderately informative is mitigated. However, the homogenous variance assumption is typically unrealistic because the heterogeneity variances are likely different across treatment comparisons [5,6,15]. As a result, 95% credible intervals may not maintain their nominal coverage, and treatment rank probabilities may be distorted [10,15]. Of course, when employing weakly informative variance priors, relaxing the homogeneous variance assumption may be equally problematic due to a reduction in precision for estimating heterogeneity across treatment comparisons.

There are a number of approaches for eliciting or constructing informative variance priors in random-effects MTCs. Further, there are a number of possible heterogeneity variance structures under which weakly informative variance priors can be employed. To date, no comparison of the available informative and weakly informative approaches is available of their relative performance. In this article we review and compare six random-effects MTC models – four under which weakly informative variance priors are elicited, and two under which moderately informative variance priors are elicited. The four weakly informative models include the conventional homogeneous variance model, the unrestricted heterogeneous variance model, the exchangeable variances model, and the consistency variances model. The two moderately informative models are structurally based on the unrestricted heterogeneous variance model and the variance priors are either frequentistic distribution approximations from within the MTC data or distributions previously derived from a large external empirical data set. We place comparative emphasis on the homogeneous variance model since this approach is conventionally used in MTC practice. We discuss how inferences from the informative approaches as well as the other weakly informative approaches theoretically line up against inferences from the conventional homogeneous variance MTC model. We compare treatment effect estimates 95% credible intervals, heterogeneity variance estimates and posterior distributions, and treatment rank probabilities from the discussed models in two illustrative examples. MTC treatment effect and variance estimates are also compared with those from pair wise meta-analyses.

## Methods

In this section we first describe, distinguish and discuss what is meant by different degrees of information contained in the prior distributions in Bayesian MTCs. We then describe the general MTC model setup, as well as the setup for the commonly applied homogeneous variance MTC model. Lastly, we describe six approaches to modelling between-trial variances that make use of different combinations of heterogeneity variance parameterizations and priors.

### Prior information terminology

In the introduction we mentioned use of ‘non-informative’, ‘weakly informative’, and ‘moderately informative’ priors. These terms are often used vaguely or interchangeably in the literature. Below, we define, distinguish and discuss what exactly is meant in this article when priors are ‘non-informative’, ‘weakly informative’, or ‘moderately informative’.

### Non-informative priors

In this article, we define ‘non-informative priors’ as prior distributions, that carry virtually no information about the likely true value of a parameter. For example, for treatment effects measured as log odds ratios in a logistic regression model (which is the typical set up for MTCs of binary data), a normal distribution with mean zero and variance 10000 carries virtually no information about the likely true log odds ratio, and thus, constitutes a non-informative prior distribution. For a between-trial variance parameter, an example of a non-informative prior could be a gamma distribution with shape and scale parameters of both 10^-10^. It should be noted that because Bayesian analysis is typically realized by Markov Chain Monte Carlo (MCMC) sampling, which relies on prior distributions and initial sampling values being sufficiently reasonable to allow for convergence of the posterior distribution, there is a limit to how non-informative a prior can feasibly be. For example, running the MCMC sampling for a Bayesian MTC may not be feasible if a gamma distribution with shape and scale parameters of 10^-10^ is used for the between-trial variance parameter.

### Weakly informative priors

In this article, we define a ‘weakly informative’ prior as a prior distribution that carries more information than a non-informative prior, but deliberately carries smaller degree of information than is actually available. The purpose of using weakly informative priors rather than non-informative priors is typically to achieve some stabilization in the MCMC sampling and/or estimation procedure. In the context of MTCs, a typical example of a weakly informative prior for the between-trial standard deviation parameter is the conventionally used uniform distribution between 0 and 2 when data is dichotomous and treatment effects are modelled as log odds ratios (ie, modelled in a logistic regression framework). This prior carries more information than a typical non-informative variance prior (e.g., the above mentioned gamma distribution). It is well known that between-trial variances on the log odds ratio scale generally do no exceed a value of 4, and so, this knowledge is used by truncating the between-trial standard deviation to 2. It is also known that between-trial variances on the log odds ratio scale are typically smaller than 1 and closer to 0. However, this knowledge is only used partially for this prior since the probability of observing larger between-trial variance values only decreases slightly for larger values [3]. The danger with using weakly informative variance priors in MTCs is that the data is often relatively sparse, and so, a variance prior that is presumed weakly informative can easily become moderately and sometimes highly informative. For example, the expected value for the heterogeneity variance from the above unifom prior is approximately 1.33. However, in a setting where the heterogeneity variance is likely to be close to 0 (e.g., very similar trials designs and drug responses do not differ much across populations) and where only a few small trials are available to inform the variance estimation, this prior may easily upward bias the heterogeneity variance estimate, and thus, create artificially wide credible intervals.

### Moderately informative priors

In this article, we define a ‘moderately informative’ prior as a prior distribution that carries a distinguishable and larger degree of information than a weakly informative prior. The purpose of using a moderately informative prior is to either fully or partially mix prior (external) knowledge about one or more parameters with the data. To this end, the data still plays an important role. One example of a moderately informative prior is use of observational data about the magnitude of one or more comparative treatment effects. For example, if observational studies have suggested that one novel treatment exhibits a 25% reduction of symptoms over another novel treatment, one can use this evidence to produce a mean parameter value (treatment effect) in the prior distribution and subsequently elicit a variance that corresponds to the weight and confidence one is willing to put in this value. Another example of moderately informative priors in the MTC framework is the use of empirical evidence on the distribution of between-study variance estimates across published meta-analyses. This is also the last of the six heterogeneous variance approaches considered in this article, and will be illustrated below.

### General MTC model set up

For this manuscript we describe MTC models of binary data. However, the modelling concepts are easily extended for other types of data such as count data and continuous data [16]. For simplicity, we also assume that all trials included in an MTC are 2-arm trials. Multi-arm trials necessitate modelling of correlations between treatment comparisons with a common comparator. We refer to previous papers for detailed description of this issue [10,13].

In the binary data setting, a commonly used effect measure in MTCs is the odds ratio (OR). For each treatment comparison, odds ratios are typically estimates with a logistic regression model that simultaneously links the trial-arm odds and the treatment comparison odds ratios. Letting *k* denote the number of trials and *T* the number of treatments in a network, and letting *t=1,…T* indicate the treatment in focus and *j=1,…k* the trial in focus, then the following main distributional and deterministic relationships make up the core of the MTC model in the Bayesian framework

(1) $$\begin{array}{c} r_{\mathrm{jt}}∼bin\left(n_{jt}p_{jt}\right)\\ \mathrm{logit}\left(p_{jt}\right)=\mu_{jt}+\delta_{jtb}1_{\left[t>b\right]}\\ \delta_{jtb}∼N\left({d_{tb,}}^{.}{\sigma_{tb}}^{2}\right)\\ d_{tb}=d_{tl}-d_{bl} \end{array}$$

Where *p*_*jt*_ is the probability of an event in trial *j* under treatment *t*, and *r*_*jt*_ and *n*_*jt*_ are the number of events and the number of patients in the corresponding treatment arm; *μ*_*jb*_ is the log odds of having an event in the control arm (i.e., with ‘baseline treatment’ *b*) in trial *j*; *δ*_*jtb*_ is the log odds ratio of treatment *t* relative to treatment *b* in trial *j*, *d*_*tb*_ is the ‘true’ overall treatment effect of *t* relative to *b*, and *σ*_*tb*_^*2*^ is the corresponding between-trial variance. The last equation represents the ‘consistency’ assumption, which is necessary for all MTC models, and dictates that any expected relative treatment effect of a direct (head-to-head) evidence source is equal to the corresponding expected relative treatment effect of an indirect evidence source. In other words, the consistency assumptions dictates that the results from direct and indirect sources of evidence should not differ beyond the play of chance.

In the above, the control arm (baseline) log odds parameters *μ*_*jb*_ are treated as nuisance parameters, and assigned non-informative normal distribution priors with mean 0 and very large variances, typically of 1000 or 10000. For *b*=1, the overall log odds ratios *d*_*tb*_ (i.e., the treatment effect of *t*) are also assigned non-informative normal distribution priors with mean 0 (representing no effect) and large variances, typically of 1000 or 10000.

### MTC models with weakly informative variance priors

#### The homogeneous variance model

Under the homogenous variance MTC model the assumption is made that all between-trial variances are equal. That is, strictly speaking we assume *σ*_*tb*_^2^ = *σ*^2^ for all treatment comparisons *t* versus *b*, or specifically, that the between-trial variance for all treatment comparisons is equal to *σ*^*2*^.

Typically a weakly informative prior is assigned to *σ* (the between-trial standard deviation) under the homogeneous variance model. Although a number of weakly informative variance priors have been used throughout the MTC literature (e.g., gamma distribution or half-normal), the most commonly used variance priors are weakly informative uniform distributions between 0 and 2 or between 0 and 10 [13,16].

#### The unrestricted heterogeneous variances model

Under the heterogeneous variance MTC models, all between-trial variances are allowed to take on different values. The *unrestricted heterogeneous variances model* places no structural restrictions on the heterogeneity variances. Under this model, weakly informative priors can be assigned to each of the between-trial variance parameters *σ*_*tb*_^*2*^. Conventionally, one would make use of the uniform distribution from 0 to 2 or from 0 to 10 as prior distributions for the between-trial standard deviations. The heterogeneous variance model with such priors is typically referred to as the unrestricted heterogeneous variance model.

Theoretically, this model is advantageous due to its high flexibility in modelling heterogeneity variances. In practice, however, this model is often sub-optimal because many comparisons are typically only informed by a few trials, and thus, the estimation of between-trial variances (i.e., their posterior distributions) is very imprecise. The below four Bayesian modelling approaches are modifications of the unrestricted heterogeneous variance model that apply different parameter value constraints or moderately informative prior distributions to optimize the estimation of the between-trial variance parameters.

#### The exchangeable variances model

One approach to gaining precision for the between-trial variance estimation is to ‘meet in the middle’ between the homogeneous and heterogeneous variances models by assuming that the between-trial variances are exchangeable. That is, one can assume that the between-trial variances are random samples from a common between-trial distribution, thus allowing them to borrow strength from each other [2]. In particular, one would assume some ‘common precision parameter, *σ*, and then sample between trial variances from any treatment comparison *t* vs *b* from a truncated *t-distribution* with *df* the degrees of freedom (the number of trials for the comparison of treatment *t* vs *b* minus 1)

(2) $$\sigma_{tb}∼t\left(0,d\;f\right),\sigma \cdot \cdot \cdot \sigma_{tb}\geq 0$$

Here we assign a weakly informative prior distribution to the ‘common’ between-trial variance corresponding to the ‘common precision’, (1/*σ*) ~ U(0,2). The prior distributions for the individual between-trial variances, *σ*_*tb*_^*2*^, can be thought of as weakly informative due to the reliance on the ‘common variance’ parameter and the degrees of freedom. We refer to this approach as the *exchangeable variances MTC model*.

Theoretically, the exchangeable variances MTC model gains the best of two worlds. It gains precision by borrowing strength from the common variance assumption, but it retains flexibility in allowing for differing between-trial variances. In practice, however, this model may not perform optimally when the between-trial variances differ considerably across comparisons. This is because the assumption of a common variance ties all individual between-trial variances probalistically to some central tendency, in which case heterogeneity parameters that are truly not close to the central tendency will be inaccurately estimated. Arguably, the exchangeable variance approach may work best in situations where 1) the interventions being investigated in the MTC are all similar (e.g., of the same drug class or solely pharmacotherapies); and 2) the study designs and patient eligibility criteria are fairly comparable.

#### The heterogeneous variances model using second order consistency inequalities

Another approach to gaining precision but retaining flexibility in modelling of heterogeneous variances is to re-parameterize the variance structure in order to ensure that the property of *consistency* also holds for the between-trial variance (and between-comparison correlation) parameters [10]. The consistency relationship for variances is as follows. For any three treatments *b, x, and y*, we assume consistency. That is, for the three corresponding (mean) comparative treatment effects *d*_*yx*_, *d*_*yb*_, and *d*_*xb*_, we assume that

(3) $$d_{yx}=d_{yb}-d_{xb}$$

This equation is also sometimes referred to as the *first order consistency equation*. Taking the variances of each side of the above equation we have

(4) $${\sigma_{yx}}^{2}={\sigma_{yb}}^{2}+{\sigma_{xb}}^{2}-2{\rho_{yx}}^{b}\sigma_{yb}\sigma_{xb}\leq {\sigma_{yb}}^{2}+{\sigma_{xb}}^{2}$$

Where *σ*_*yx*_^*2*^*, σ*_*yb*_^*2*^ and *σ*_*xb*_^*2*^, are the variances of *d*_*yx*_, *d*_*yb*_, and *d*_*xb*_, respectively, and *ρ*_*yx*_^*b*^ is the correlation between *d*_*yb*_, and *d*_*xb*_. The above equation implies a *second order* consistency triangle inequality

(5) $$\left|{\sigma_{yb}}^{2}-{\sigma_{xb}}^{2}|\;\leq \;{\sigma_{yx}}^{2}\leq \;|{\sigma_{yb}}^{2}+{\sigma_{xb}}^{2}\right|$$

Where |*x*| denotes the absolute value of any variable, *x*. This inequality can be incorporated in the model to restrict the variance and correlation parameters to plausible possible values and allow for better adherence to consistency. However, incorporating the consistency triangle inequality in the conventional heterogeneous variance MTC model can create serious difficulties in assigning appropriate priors. To solve this issue, Lu and Ades proposed a re-parameterization of the heterogeneous variance model in which each between-trial variance parameter would be represented by the sum of variances of the two involved treatment arms minus the corresponding covariance [10]. The resulting covariance matrix is represented as the product of variance vectors and a correlation matrix, where the correlation matrix is constructed via a Cholesky decomposition using spherical coordinates to allow for weakly informative priors. We refer to the paper by Lu and Ades for the mathematical details [10]. For the remainder of this paper we refer to the above approach as the *consistency variances MTC model.*

Theoretically, the consistency variances model is optimal in that it largely retains the flexibility of the unrestricted variances model, and additionally restricts variances in alignment with and borrows strength from the seminal assumption of consistency. In practice, the consistency triangular inequality may not hold within the available data since between-trial variance estimates (and posterior distributions) may fluctuate and differ due to the play of chance [17], time-dependent biases [18], and binary event rates [19]. Incorporating the consistency triangular inequality imposes an adjustment to the variances if the inequality is not met within the data, but there is no guarantee that this adjustment is in the right direction.

### MTC models with moderately informative variance priors

Considering the limitation of the above models, one could argue that random-effects MTCs incorporating sensible moderately informative variance priors constitute a viable alternative. Below we propose two sensible approaches for obtaining and eliciting informative variance priors in random-effects MTCs.

#### Using frequentist within-data approximate distribution as priors

Informative variance priors should aid in ensuring that the estimation of between-trial variances is directed with appropriate probability mass to plausible intervals of possible values. It therefore seems reasonable to require that variance estimates and their posterior probability distributions should be directed towards the values one would have obtained in separate pair wise meta-analysis, and vice versa [11]. We therefore put forward, that the probability distributions for the between-trial to variance estimated from the available data in a frequestist framework could readily be used as informative variance priors in MTCs. While a number of methods are available for estimating variance distributions, we particularly consider the approximate gamma distribution proposed by Biggerstaff and Tweedie [20], albeit in a modified version to fit MTC modeling. This frequentist approximate distribution is a location-shifted, scaled gamma distribution for the DerSimonian-Laird (DL) estimator, *σ*_*DL*_^*2*^, based on the relationship between this estimator and Cochran’s *Q* (test for heterogeneity), *σ*_*DL*_^*2*^ = (*Q*-(*k*-1))/(*S*_*1*_ – (*S*_*2*_*/S*_*1*_)), where *k* is the number of trials, *S*_*1*_ is the sum of trial weights (ie, inverse variances) and *S*_*2*_ is the sum of squared trial weights [21]. With respect to the two treatments being compared, x and y, the approximate gamma distribution of *Q* and its parameters are given

(6) $$\begin{array}{l} Q_{yx}∼\Gamma \left(r_{yx,}\lambda_{yx}\right)\\ r_{yx}=E{\left(Q_{yx}\right)}^{2}/Var\left(Q_{yx}\right)\\ \lambda_{yx}=E\left(Q_{yx}\right)/Var\left(Q_{yx}\right) \end{array}$$

Where *E(Q*_*yx*_*)* and *Var(Q*_*yx*_*)* is the expected value and variance of *Q*_yx_. We refer to the paper by Biggerstaff and Tweedie for the approximate deterministic expressions of *E(Q*_*yx*_*)* and *Var(Q*_*yx*_*)*[20].

While the approximate distribution for *σ*_*DL*_^*2*^ for any comparison is a candidate as an informative variance prior, it does have some undesirable limitations in the context of Bayesian analysis. First, *σ*_*DL*_^*2*^ can yield negative estimates and will in this case be truncated to 0 [21]. If used as a variance prior in the Bayesian framework, this property may create a bi-modality on the posterior distribution. Such a bi-modality may increase the time to convergence of the Markov Chain Monte Carlo (MCMC) sampling and result in poor model fits (ie, large deviance information criterion, DIC). Another issue is the well-known tendency of *σ*_*DL*_^*2*^ to underestimate the between-trial variance [7,8,22]. To avoid these issues, we propose to use a consistently positive estimator proposed by Hartung and Makambi (HM) [23]. In contrast with the DL estimator, which is derived as a 1^st^ order method of moments estimator, the HM estimator is a 2^nd^ order method of moments based estimator and has the following expression

(7) $${\sigma_{HM}}^{2}=Q\left((2\left(k-1\right)+Q\right)\left(S_{1}-\left(S_{2}/S_{1}\right)\right)$$

The HM estimator is consistently positive and has been shown to yield accurate and precise estimates of the between-trial variance [7,9,23]. HM is a function of *Q*, and thus, by incorporating the prior distribution of *Q* in the WinBUGS code and subsequently deriving *σ*_*HM*_^*2*^ via its original expression, the shortcomings of the DL approach are circumvented.

The above proposed approach for obtaining and eliciting informed variance priors is either optimal or sub-optimal depending on the assumptions one is willing to make. By informing variance estimation with prior distributions corresponding to the expected likelihood in a frequentist analysis, one imposes a ‘2-stage’ estimation process that lets the Bayesian MCMC sampling ‘concentrate’ on the estimation of treatment effects. An analogous process was recently proposed in the purely frequentist framework [11]. The informed variance prior approach, however, is sub-optimal if one is not willing to believe the frequentist variance likelihoods and prefers to incorporate additional uncertainty around variance estimation. Further, approxi-mating the heterogeneity variance distributions as suggested above, may be work intensive.

#### Heterogeneous variances using empirically derived informative priors

A simpler and more general approach to incorporating informed variance priors is to borrow strength from external empirical evidence. Turner et al. reviewed 14886 Cochrane Database meta-analyses including a total 77237 trials and approximated the empirical distribution of the between-trial variance categorized by type of outcome (mortality, semi-objective and subjective), type of intervention, and field of medicine [6]. The mean and variance parameter values for log-normal distributions were estimated by category [6]. These empirically derived log-normal distributions can readily be used as moderately informative variance priors under the unrestricted heterogeneous variance model. For example, Turner et al. empirically approximated the heterogeneity variance distribution for meta-analyses comparing pharmacological interventions on subjective outcomes (e.g., dichotomous biomarker outcome) to a log-normal distribution with mean −2.34 and variance 1.62 [2]. In an MTC comparing only pharmacological interventions on a subjective outcome (as is the case in illustrative example 1), one can then elicit this log-normal distribution for all heterogeneity variance parameters instead of the conventional weakly informative uniform distribution.

This informative variance approach is relatively straightforward to apply. The already empirically approximated priors have general applicability due to the sample size of the empirical study from which they originated. However, to the extent other factors than the ones explored by Turner et al. determine the likely degree and distribution of heterogeneity variance, the approach may not produce optimal variance estimation.

## Results

**Figure 1:**
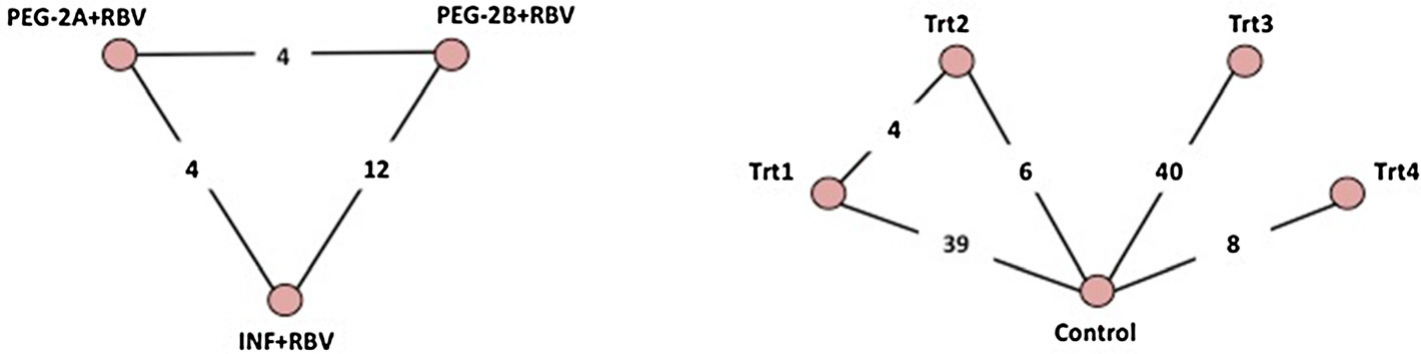
**Presents the treatment networks with the number of trials informing each treatment comparison in our two illustrative examples.** The treatment network on the left is the network for our first illustrative example. The treatment network on the right side is the network for our second illustrative example. The circles represent the treatments in the network, the lines represent the comparisons where head-to-head (direct) evidence is available, and the numbers in the lines present the number of randomized clinical trials available per comparison. Abbreviations: PEG-2A (Peginterferon-2a); PEG-2B (Peginterferon-2b); INF (Interferon), RBV (Ribavirin); Trt (Treatment).

We applied the above considered models and priors to two MTC data sets of differing size and complexity to illustrate the performance. The treatment networks for our two examples are presented in Figure 1. We compared the inferences from the five described heterogeneous variance MTC models with the homogeneous variance MTC model and with reference to the heterogeneity estimates obtained from pair wise meta-analysis. In particular, we compared 1) the model fit (using the deviance information criterion (DIC)) as well as the estimates and posterior distributions of the between-study heterogeneity variances; 2) the magnitude, direction and significance of each treatment comparison; and 3) the ranking of the treatments in terms of probabilities of being the best treatment.

The DIC is a measure of model fit computed from the likelihood function with a penalty for complexity [24]. The complexity is measured as the ‘effective number of parameters’, which is abbreviated ‘pD’ [24]. The DIC is similar to the AIC and BIC, and a lower value means a better fit [24]. The probability of ‘being the best treatment’ is derived as the probability of being the largest odds ratio among MCMC simulations from the posterior distribution.

We compared the heterogeneity variances from all MTC models with the DerSimonian-Laird and Hartung-Makambi estimates from pair wise meta-analyses, as well as with the Bayesian pair wise meta-analysis estimates. Considering the pair wise heterogeneity variance estimates as the bench mark, we then assessed the extent to which observed differences in inferences between MTC models could be explained by poor estimation of between-study heterogeneity variances and their posterior distributions.

All Bayesian MTC models were carried out in WinBUGS v.1.4.3 [25]. Convergence of Markov Chain Monte Carlo simulation was assessed using the Brooks-Gelman-Rubin criteria using 3 chains, and based on the findings of the convergence analysis, a burn-in of 20000 iterations was used for all MTC analysis. Similarly, MTC model inferences were based on 20000 iterations following the burn-in period. Frequentist meta-analyses were carried out in *R* v.2.14 [26].

### Illustrative example 1

In our first example, we use data from two Cochrane Database systematic reviews on interventions for treating hepatitis C [27,28]. The MTC data set is a simple fully connected treatment network of the three interventions: PegInterferon alpha-2a plus Ribavirin (PEG-2a+RBV), PegInterferon alpha-2b plus Ribavirin PEG-2b+RBV), and standard Interferon + Ribavirin (INF+RBV) (see Figure 1a). The population is limited to treatment-naïve patients and excludes patients with co-infections (e.g., HIV). We use the meta-analysis data for the conventionally used surrogate efficacy measure sustained virologic response (SVR).

In this data set, each of the three treatment comparisons is informed by a comparable amount of evidence. In particular, the comparison of PEG-2a+RBV and INF+RBV includes 4 trials and 1197 patients, the comparison of PEG-2b+RBV and INF+RBV includes 12 trials and 2750 patients, and the comparison of PEG-2a+RBV and PEG-2b+RBV includes 6 trials and 2994 patients. The trials in the three comparisons (pairwise meta-analyses) each incurred different degrees of heterogeneity (e.g., DerSimonian-Laird between-trial variance estimates of 0.64, 0.00, and 0.04). This suggests a need for modelling the between-trial variances as heterogeneous in the MTC model, which makes this data set a good candidate for how well the heterogeneous variance MTC models perform in this context and how they measure up against the conventional homogeneous variance model. For the ‘empirically informed variances’ model we used a log-normal distribution with mean −2.34 and variance 1.62 [2] because all interventions being compared are pharmacological and the outcome, SVR, is a dichotomous biological marker, which fits under ‘subjective outcome’ definition by Turner et al. [6].

**Figure 2:**
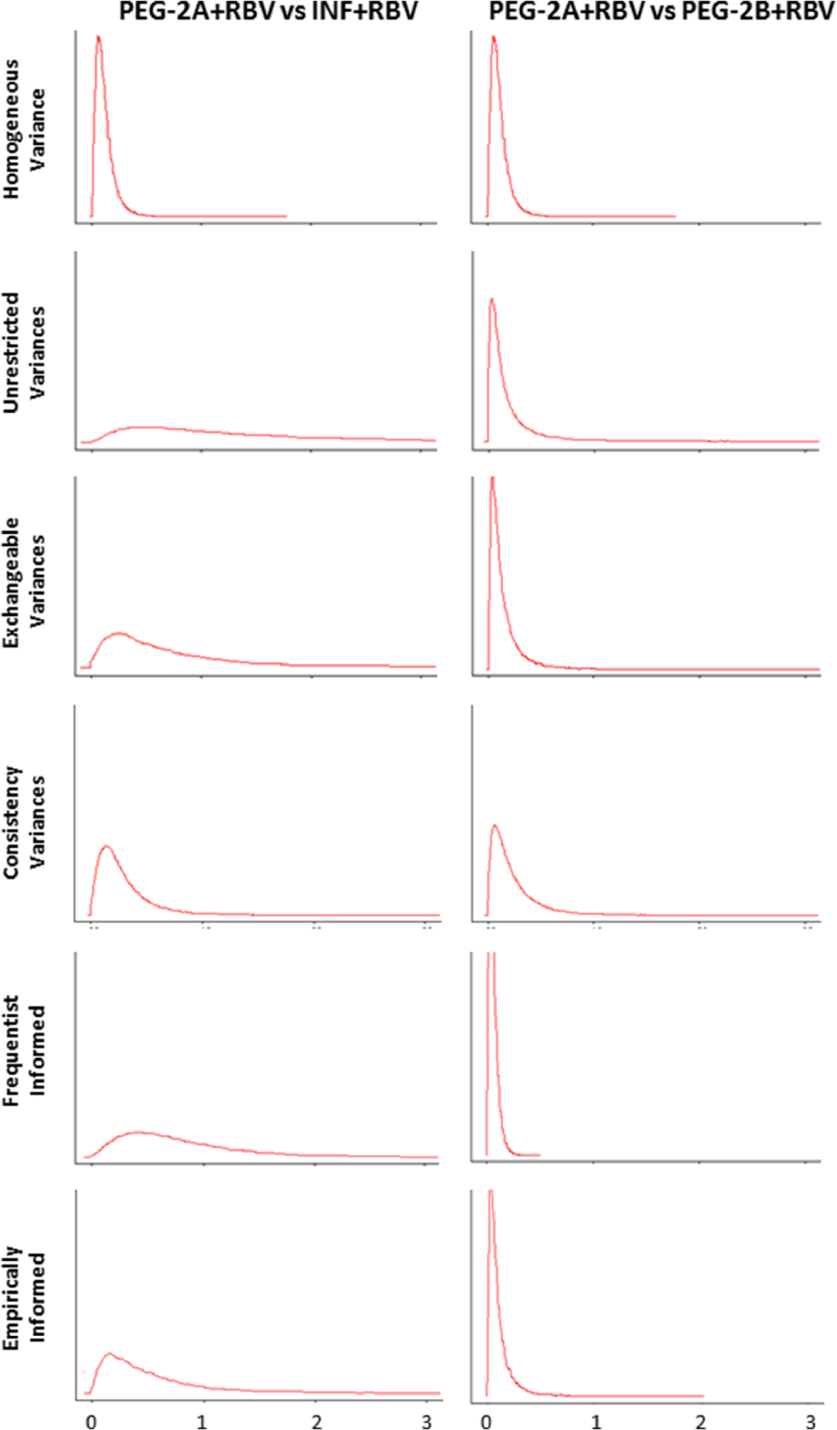
**Presents the posterior distributions of the between-trial variance parameters in the first illustrative example under the six employed MTC models: the homogeneous variance model (row 1); the unrestricted variances model (row 2); the exchangeable variances model (row 3); the consistency variances model (row 4); the frequentistically informed variances model (row 5); and the empirically informed variances model (row 6).** The two presented comparisons are: peginterferon-2a (PEG-2A) vs Interferon (INF) (column 1), and Peginterferon-2a (PEG-2A) vs Peginterferon-2b (PEG-2B) (column 2). The comparison of PEG-2B vs INF was selective excluded due to the posterior variance distributions being more similar across the five heterogeneous variance approaches.

As expected, the homogeneous variance MTC models yielded a worse model fit than the heterogeneous variance MTC models according to the DIC (Table 1). The informed variance model based on frequentist approximate distributions yielded the best model fit according to the DIC. The remaining four heterogeneous variance models yielded comparable DICs. Comparison of the ‘common’ between-trial variance estimate with the frequentist estimates as well as the estimates from the five heterogeneous variances MTC models strongly suggests that the ‘homogeneous variance’ assumption is both strongly violated and will result in an unrealistic between-trial variance estimates for most (if not all) comparisons (Table 1). Among the five heterogeneous variances MTC model, the informed variances model based on frequentist approximate distributions produced variance estimates closest to the frequentist ones and had the posterior variance distributions with the highest precision (Figure 2). The empirically informed variances model had the second highest posterior distribution precision, the consistency variances model third, the exchangeable variances model fourth, and lastly the unrestricted variances model fifth (Figure 2).

**Table 1:** Between-trial variance estimates and model fit statistics from the considered models and priors in the first illustrative example on hepatitis C treatments for achieving sustained virological response (SVR)

	**Between-trial variance estimates**			**Model fit statistics**	
**Models**	**PEG-2A+RBV vs INF+RBV**	**PEG-2B+RBV vs INF+RBV**	**PEG-2B+RBV vs PEG-2A+RBV**	**pD**	**DIC**
*Random-effects pair wise meta-analysis*					
Frequentist (DerSimonian-Laird)	0.642	0.000	0.036	--	--
Frequentist (Hartung-Makimbi)	0.580	0.017	0.021	--	--
Bayesian (weakly informative)	0.700	0.018	0.077	--	--
Bayesian (frequentist informed)	0.422	0.001	0.038	--	--
Bayesian (empirically informed)	0.091	0.024	0.052	--	--
*Random-effects MTC models*					
*Weakly informed variance models*					
*Homogeneous variance model*	0.097	0.097	0.097	33.3	283.2
Unrestricted variances	1.046	0.017	0.103	32.8	277.7
Exchangeable variances	0.510	0.016	0.083	32.6	278.6
Consistency variances structure	0.225	0.024	0.164	32.9	280.2
*Moderately informed variance models*					
Frequentist informed priors	0.677	0.011	0.047	30.9	275.6
Empirically informed priors	0.368	0.026	0.076	32.4	278.4

Abbreviations: PEG-2A (Peginterferon-2a); PEG-2B (Peginterferon-2b); INF (Interferon), RBV (Ribavirin).

For the comparison between peginterferon-2a and interferon and the comparison between the two peginterferons, the homogeneous variance model has narrower 95% credible intervals that all other heterogeneous variance models, except for the informed variance model based on frequentist approximate distributions (see Table 2). For the comparison between peginterferon-2a and interferon, the homogeneous variance model yielded a comparably wider 95% credible interval (see Table 2). The unrestricted variances model had the widest credible intervals among the heterogeneous variances models, and the informed variances model based on frequentist approximate distributions had the narrowest credible intervals. Because this network only included three treatments we did not calculate treatment rank probabilities.

**Table 2:** Odds ratios and 95% confidence/credible intervals for the three comparisons from the considered models and priors in the first illustrative example on hepatitis C treatments for achieving sustained virological response (SVR)

**Model**	**PEG-2A+RBV vs INF+RBV**	**PEG-2B+RBV vs INF+RBV**	**PEG-2B+RBV vs PEG-2A+RBV**
*Random-effects pair wise meta-analysis*			
Frequentist (DerSimonian-Laird)	3.63(1.51-8.73)	1.30(1.11-1.52)	1.38(1.07-1.79)
Frequentist (Hartung-Makimbi)	3.60(1.56-8.34)	1.35(1.11-1.64)	1.38(0.36-3.24)
Bayesian (weakly informative)	NA*	1.36(1.10-1.73)	1.38(0.94-2.22)
Bayesian (frequentis informed)	NA*	1.30(1.11-1.55)	1.40(0.86-2.47)
Bayesian (empirically informed)	NA*	1.36(1.10-1.74)	1.38(1.02-1.99)
*Random-effects MTC models*			
*Weakly informed variance models*			
*Homogeneous variance model*	2.42(1.75-3.60)	1.53(1.19-2.03)	1.58(1.18-2.26)
Unrestricted variances	2.11(1.40-3.57)	1.38(1.13-1.79)	1.50(1.06-2.53)
Exchangeable variances*	2.17(1.48-3.43)	1.40(1.15-1.79)	1.53(1.11-2.36)
Consistency variances structure	2.39(1.63-3.80)	1.42(1.16-1.86)	1.67(1.17-2.68)
*Moderately informed variance models*			
Frequentist informed priors	2.04(1.45-2.93)	1.38(1.15-1.69)	1.46(1.12-2.05)
Empirically informed priors	2.23(1.54-3.40)	1.44(1.16-1.83)	1.54(1.13-2.29)

* The MCMC simulation did not converge for the log odds ratio parameter (within the first 1.000.000 runs), and thus did not produce meaningful results.

Abbreviations: PEG-2A (Peginterferon-2a); PEG-2B (Peginterferon-2b); INF (Interferon), RBV (Ribavirin).

### Illustrative example 2

Our second example data set is a larger, more diverse treatment network including four pharmacological interventions (Trt1, Trt2, Trt3, and Trt4) and a control for cessation of a harmful behaviour (See Figure 1b) [15]. In this example the outcome of interest is taken at 6 months follow-up. The included studies all enrolled participants at initiation of therapy. Each of the four interventions had been compared to control, and the first two had been compared to each other. The amount of evidence differed across comparisons. In particular, Trt1 versus placebo was informed by 39 trials and 16674 patients, Trt2 versus placebo was informed by 6 trials and 3222 patients, Trt3 versus placebo was informed by 40 trials and 10682 patients, and the Trt4 versus placebo was informed by 8 trials and 3678 patients, and lastly, Trt 2 vs Trt 1 was informed by 4 trials and 2330 patients. These five head-to-head comparisons (pairwise meta-analyses) incurred only moderately different degrees of heterogeneity, except for Trt3 versus placebo where little to no heterogeneity was detected (see Table 3). This suggests the homogeneous variance model may not perform too poorly. However, the situation still raises uncertainty about which model is most suitable and therefore warrants modelling with a proposed heterogeneous variance models for the purpose of identifying the best fit (and thus most valid inferences). For the ‘empirically informed variances’ model we used a log-normal distribution with mean −3.02 and variance 1.85 [2] because all placebo comparisons and a log-normal distribution with mean −3.23 and variance 1.88 [2] for comparison of active interventions, since all interventions being compared are pharmacological and the outcome, cessation to a harmful behavious, fits under the ‘semi-objective outcome’ definition by Turner et al [6].

**Table 3:** Between-trial variance estimates (posterior distribution median) for the comparisons that were also informed by head-to-head evidence in the treatment network in the second illustrative example

	**Between-trial variance estimates**					**Model fit statistics**	
**Models**	**Trt1 vs Placebo**	**Trt2 vs Placebo**	**Trt3 vs Placebo**	**Trt4 vs Placebo**	**Trt2 vs Trt1**	**pD**	**DIC**
*Random-effects pair wise meta-analysis*							
Frequentist (DerSimonian-Laird)	0.086	0.110	0.016	0.075	0.106	--	--
Frequentist (Hartung-Makimbi)	0.083	0.103	0.040	0.072	0.112	--	--
Bayesian (weakly informative)	0.100	0.371	0.023	0.103	0.334	--	--
Bayesian (frequentist informed)	0.087	0.121	0.036	0.067	0.093	--	--
Bayesian (empirically informed)	0.088	0.110	0.021	0.054	0.059	--	--
*Random-effects MTC models*							
*Weakly informed variance models*							
*Homogeneous variance model*	0.078	0.078	0.078	0.078	0.078	138.8	1229.1
Unrestricted variances	0.100	0.469	0.023	0.104	0.214	138.3	1229.9
Exchangeable variances*	0.092	0.226	0.009	0.066	0.047	133.7	1232.2
Consistency variances structure	0.091	0.172	0.033	0.075	0.133	136.6	1230.0
*Moderately informed variance models*							
Frequentist informed priors	0.087	0.172	0.036	0.069	0.064	135.6	1226.4
Empirically informed priors	0.087	0.199	0.020	0.054	0.042	133.8	1229.5

* The’average’ variance was 0.173.

Abbreviations: DIC (Deviance information criterion); pD (effective number of model parameters); Trt (Treatment).

**Figure 3:**
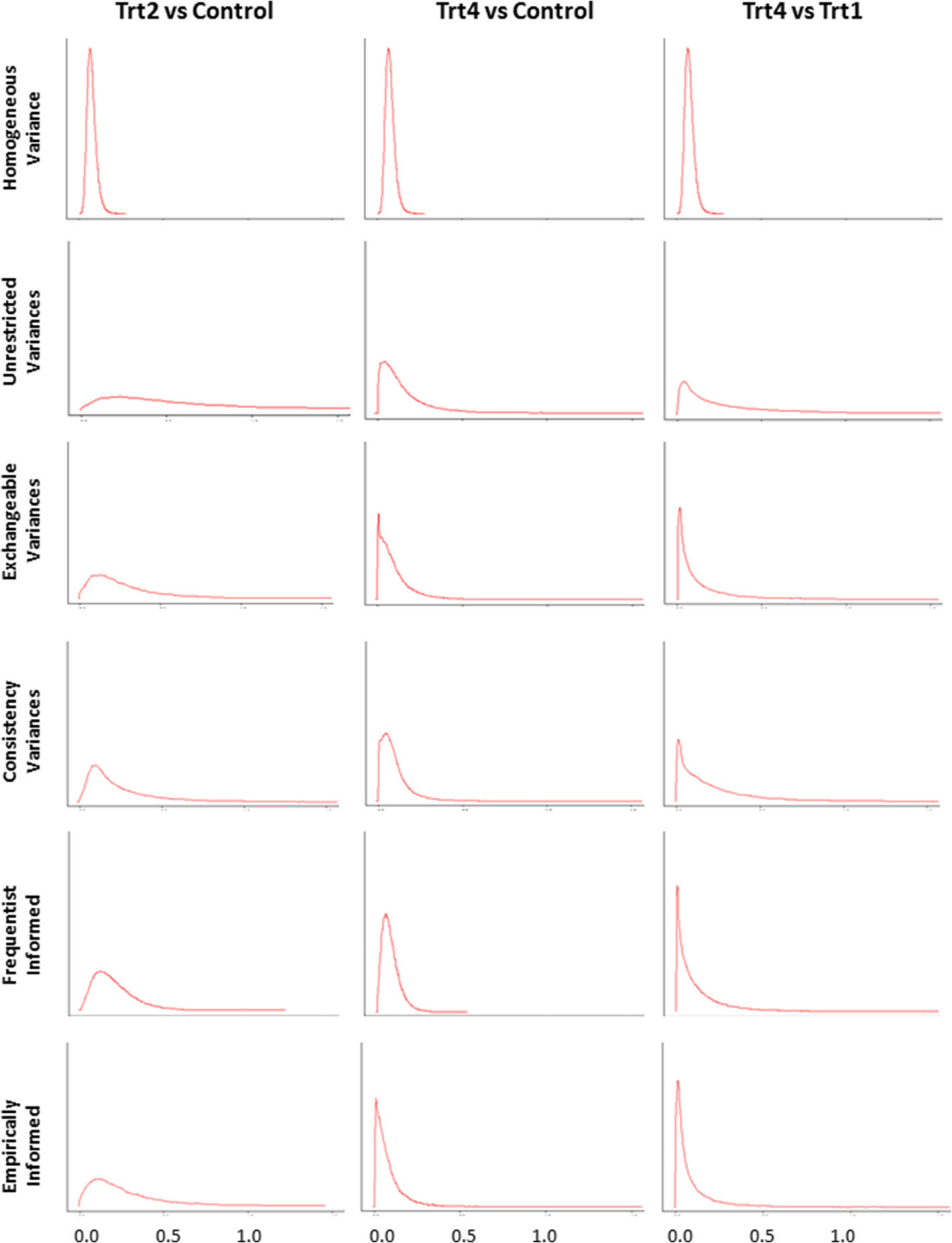
**Presents the posterior distributions of the between-trial variance parameters in the second illustrative example under the six employed MTC models: the homogeneous variance model (row 1); the unrestricted variances model (row 2); the exchangeable variances model (row 3); the consistency variances model (row 4); the frequentistically informed variances model (row 5); and the empirically informed variances model (row 6).** The three presented comparisons are: Treatment 2 (Trt2) versus control (column 1); treatment 4 (Trt2) versus Control; and Trt4 versus Trt1. The remaining comparisons were selective excluded due to the posterior variance distributions being more similar across the five heterogeneous variance approaches.

According to the DIC, the informed variances model based on the frequentist approximate variance distributions yielded the best model fit (Table 3). The homogeneous variance model and the remaining four heterogeneous variances models yielded similar model fits according to the DIC (Table 3). Comparison of the ‘common’ between-trial variance estimate with the frequentist estimates as well as the estimates from the five heterogeneous variances MTC models suggests that the ‘homogeneous variance’ assumption is mildly to moderately violated. Among the five heterogeneous variances MTC model, the consistency variance model and the informed variances model using frequentist approximate distributions produced estimates closest to the frequentist ones. The exchangeable variance model and the empirically informed variances model also produced seemingly reliable variance estimates. Again, the informed variance model using frequentist approximate distributions had the highest posterior distribution precision (Figure 3). The empirically informed variances model had the second highest posterior distribution precision, the consistency variances model third, the exchangeable variances model fourth, and lastly the unrestricted variances model produced the most imprecise posterior distributions. Figure 3 Presents the posterior distributions of the between-trial variance parameters in the second illustrative example under the six employed MTC models: the homogeneous variance model (row 1); the unrestricted variances model (row 2); the exchangeable variances model (row 3); the consistency variances model (row 4); the frequentistically informed variances model (row 5); and the empirically informed variances model (row 6). The three presented comparisons are: Treatment 2 (Trt2) versus control (column 1); treatment 4 (Trt2) versus Control; and Trt4 versus Trt1. The remaining comparisons were selective excluded due to the posterior variance distributions being more similar across the five heterogeneous variance approaches.

The treatment effect estimate and 95% credible interval for Trt2 were considerably affected by the variance assumption, and thus, so were indirect comparisons between Trt2 versus other interventions (Table 4). The treatment effect estimate of Trt2 versus placebo was smallest with the homogeneous variance model, and the 95% credible intervals were narrow compared with those of the heterogeneous variances models. These differences considerably impacted treatment rank probabilities. While Trt1 and Trt3 consistently received very low rank probabilities (e.g., 0.5% chance of being the best), the probability of Trt2 versus Trt4 being the best treatment varied from 71.3% versus 28.2% with the homogeneous variance model to informed variance model to 43.2% versus 56.8% with the unrestricted variance model (see Table 5).

**Table 4:** Odds ratios and 95% confidence/credible intervals for the four placebo comparisons and two select active intervention comparisons in the second illustrative example

**Model**	**Trt1 vs Placebo**	**Trt2 vs Placebo**	**Trt3 vs Placebo**	**Trt4 vs Placebo**	**Trt2 vs Trt1**	**Trt4 vs Trt2**
*Random-effects pair wise meta-analysis*						
*DerSimonian-Laird*	1.94(1.67-2.24)	2.11(1.42-3.13)	1.78(1.60-1.97)	2.86(2.21-3.71)	1.90 (1.17-3.09)	--
*Hartung-Makimbi*	1.94(1.68-2.23)	2.09(1.42-3.09)	1.77(1.57-2.00)	2.86(2.21-3.70)	1.91 (1.16-3.13)	--
*Bayesian (non-informative)*	1.98(1.70-2.31)	2.38(1.30-5.82)	1.80(1.61-2.02)	2.89(2.08-4.06)	1.97 (0.66-8.88)	--
*Bayesian (frequentist informed)*	1.97(1.71-2.28)	2.16(1.48-3.68)	1.80(1.60-2.02)	2.89(2.23-3.77)	1.79 (1.17-3.55)	--
*Bayesian (empirically informed)*	1.97(1.71-2.28)	2.13(1.47-3.98)	1.80(1.61-2.03)	2.89(2.20-3.80)	1.84 (1.17-3.53)	--
*Random-effects MTC models*						
*Weakly informed variance models*						
*Homogeneous variance model*	1.91(1.67-2.19)	2.59(1.97-3.50)	1.80(1.57-2.08)	2.90(2.23-3.79)	1.36 (1.02-1.84)	1.11(0.74-1.63)
Unrestricted variances	1.95(1.69-2.28)	3.05(1.84-5.50)	1.81(1.62-2.01)	2.90(2.07-4.07)	1.56 (0.94-2.75)	0.94(0.49-1.74)
Exchangeable variances*	1.93(1.67-2.26)	2.89(1.98-4.43)	1.78(1.56-1.99)	2.89(2.17-3.86)	1.50 (1.03-2.26)	1.01(0.59-1.58)
Consistency variances structure	1.93(1.66-2.23)	2.80(1.92-4.69)	1.80(1.60-2.02)	2.90(2.18-3.86)	1.45 (0.99-2.40)	1.03(0.58-1.66)
*Moderately informed variance models*						
Frequentist informed priors	1.93(1.68-2.22)	2.80(1.98-4.10)	1.80(1.60-2.03)	2.90(2.22-3.78)	1.46 (1.02-2.11)	1.05(0.66-1.61)
Empirically informed priors	1.93(1.67-2.23)	2.84(2.00-4.26)	1.80(1.61-2.02)	2.91(2.22-3.81)	1.48 (1.00-2.20)	1.02(0.63-1.59)

Abbreviations: DIC (Deviance information criterion); pD (effective number of model parameters); Trt (Treatment).

**Table 5:** Treatment rankings, the probability of being the best treatment, under the considered Bayesian

**Model and prior**	**Trt1**	**Trt2**	**Trt3**	**Trt4**
*Weakly informed variances models*				
*Homogeneous variance*	0.00%	28.2%	0.00%	71.8%
Unrestricted variances	0.01%	56.8%	0.00%	43.2%
Exchangeable variances	0.01%	49.3%	0.02%	50.4%
Consistency variances structure	0.04%	45.0%	0.01%	55.9%
*Moderately informed variances models*				
Frequentist informed priors	0.00%	42.5%	0.00%	57.5%
Empirically informed priors	0.00%	46.5%	0.00%	53.5%

MTC models for the second illustrative example.

In this example, a number of reasons suggest the informed variances model based on frequentist approximate variance distributions is the more optimal choice. First, this model clearly yields the best model fit according to the DIC. Second, it produces the variance estimates closest to those of the frequentist pair wise meta-analyses. Lastly, the full MTC from which this example is borrowed, the efficacy of the considered interventions was also investigated for 1 month, 3 months, and 12 months follow-up. For these outcomes, many of the comparisons were non-significant (i.e., the 95% credible intervals included 1.00) with the homogeneous variance model despite clear statistical significance in the pair wise meta-analyses. When we used variance priors informed by frequentist approximate variance distributions, this statistical significance was recovered.

## Discussion

The variance structure in an MTC is challenging to estimate because it rests on the amount of evidence and the linkage between comparisons. A number of approaches are available, but their performance is tied with the appropriateness of the assumed linkage between comparisons, and in the Bayesian framework, the elicited variance priors. Conventional MTC models have made use of the unrealistic assumption that the between trial variances for the included comparisons are all equal [4-6,10,15]. Emerging evidence (including our examples), however, suggest this approach is sub-optimal [10,15]. Instead, there is a need to consider ‘heterogeneous variance structures’. Because the amount of evidence to reliably estimate heterogeneity variance parameters is typically sparse, some precision can be gained either by incorporating informative variance priors or by using alternative restrictive heterogeneity variance structures in connection with weakly informed variance priors. In this paper we have considered two types of informative variance priors: frequentist and empirically informed; and we considered two restrictive variance structures with weakly informative priors: the exchangeable variances approach, and the consistency variances approach.

Our examples suggest that these four approaches all allow for reliable estimation of differing between-study heterogeneity variances across comparisons, whereas the unrestricted approach often does not. To this end, these four approaches seem superior to the homogeneous variance structure model as well as the unrestricted heterogeneous variances approach. The frequentist informed approach yielded the best model fits in both example, and although further research is needed at this point, one could argue for this approach as a primary supplement to the conventional homogeneous model.

Our study offers several strengths, but also has some limitations. Our chosen illustrative examples are of different size and complexity and yield heterogeneity estimates for which the homogeneous variance assumption was violated to an extend that impacted the findings of the MTCs. Our study is also the first to compare multiple weakly and moderately informed approaches to modelling heterogeneity in MTCs. Our study, however, is by no means generalizable to all MTCs. Several treatment networks may exist or emerge in which, for example, the homogeneous variance model and some heterogeneous variance model will yield close to equal inferences about all comparative treatment effects. In this vein, it is important that authors and readers of MTCs continually pay careful consideration to the fragility of variance estimation, credible intervals and treatment rank probabilities. Another limitation is the empirical nature of this study. With empirical data we can only observe differences, but never infer definitively about the truth. In this context, simulation studies would be needed to investigate the performance of the models based on bias, precision, MSE, etc., under different scenarios and types of networks. However, we believe additional empirical studies are necessary to inform which scenarios are truly important to explore under simulation.

Appropriate modelling of heterogeneity variances in MTCs will become increasingly important over the next years. First, ‘statistical significance’ and treatment rank probabilities can be sensitive to the employed variance structure and variance priors [15]. Since regulatory agencies and clinical decision makers increasingly rely on comparative effectiveness inferences from MTCs, choosing the appropriate variance structures and priors (and necessary sensitivity analyses) also becomes increasingly important.

Further, we will likely see an increase in MTCs incorporating meta-regression or subgroup analysis to explain the observed heterogeneity by effect modification caused by some clinical covariate(s). In this vein, appropriately estimating the unexplained degree of heterogeneity for each treatment comparison is seminal to reliable estimation of the effect modification caused by some clinical covariate(s). In other words, without unbiased quantification of heterogeneity it becomes increasingly challenging to explain heterogeneity.

## Conclusions

In conclusion, MTC models using either a homogenous variance structure or weakly informative variance priors in connection with an unrestricted heterogeneous variance structure both have serious methodological shortcomings. Using informative variance priors in connection with an unrestricted variance structure or borrowing strength by assuming exchangeability or imposing consistency between heterogeneity variances, can all ensure sufficiently reliable and realistic heterogeneity estimation, and thus reliable MTC inferences. All four approaches should be viable candidates for replacing or supplementing the conventional homogeneous variance MTC model, which is currently used widely in practice.

## Competing interests

The authors declare that they have no competing interests.

## Authors’ contributions

KT drafted the first version of the manuscript, conceived the idea of the study, contributed to the design of the study, and performed all statistical analysis. LT contributed to the design of the study and writing of the manuscript. EM co-conceived the idea of the study, contributed to the design of the study, and contributed to the writing of the manuscript. All authors read and approved the final manuscript.

## Pre-publication history

The pre-publication history for this paper can be accessed here:

http://www.biomedcentral.com/1471-2288/13/2/prepub

## References

[B1] Coleman C Phung O Cappelleri J Baker W Kluger J White M Use of network meta-analysis in systematic reviews 2012 Under review: AHRQ

[B2] Gelman A Prior distributions for variance parameters in hiearchical models Bayesian Anal 2006 1 3 515

[B3] Lambert PC Sutton AJ Burton PR Abrams KR Jones DR How vague is vague? A simulation study of the impact of the use of vague prior distributions in MCMC using WinBUGS Stat Med 2005 24 15 2401 2428 10.1002/sim.2112 16015676

[B4] Thorlund K Steele R Platt R Shrier I Rapid response to Methodological problems in the use of indirect comparisons for evaluating healthcare interventions: survey of published systematic reviews’ by Song F et al BMJ 2009 10.1136/bmj.b1147 PMC2665205 19346285

[B5] Pullenayegum E An informed reference prior for between-study heterogeneity in meta-analysis of binary outcomes Stat Med 2011 30 13 10.1002/sim.4326 22020726

[B6] Turner RM Davey J Clarke M Thompson S Higgins JP Predicting the extent of heterogeneity in meta-analysis, using empirical data from the Cochrane Database of Systematic Reviews Int J Epidemiol 2012 10.1093/ije/dys041 PMC3396310 22461129

[B7] Sanchez-Meca J Marin-Martinez F Confidence intervals for the overall effect size in random-effects meta-analysis Psychol Methods 2008 13 1 31 48 18331152 10.1037/1082-989X.13.1.31

[B8] Sidik K Jonkman JN A comparison of heterogeneity variance estimators in combining results of studies Stat Med 2007 26 9 1964 81 10.1002/sim.2688 16955539

[B9] Thorlund K Wetterslev J Awad T Thabane L Gluud G Comparison of statistical inferences from the DerSimonian-Laird and alternative random-effects model meta-analyses - ana empirical assessment of 920 Cochrane primary outcome meta-analyses Res Synth Meth 2011 2 14 10.1002/jrsm.53 26061888

[B10] Lu G Ades A Modeling between-trial variance structure in mixed treatment comparisons Biostatistics 2009 10 4 792 805 10.1093/biostatistics/kxp032 19687150

[B11] Lu G Welton N Higgins JP White IR Ades A Linear inference for mixed treatment comparison meta-analysis: A two-stage approach Res Synth Meth 2011 2 18 10.1002/jrsm.34 26061599

[B12] Higgins JP Whitehead A Borrowing strength from external trials in a meta-analysis Stat Med 1996 15 24 2733 49 10.1002/(SICI)1097-0258(19961230)15:24<2733::AID-SIM562>3.0.CO;2-0 8981683

[B13] Lu G Ades AE Combination of direct and indirect evidence in mixed treatment comparisons Stat Med 2004 23 20 3105 24 10.1002/sim.1875 15449338

[B14] Lumley T Network meta-analysis for indirect treatment comparisons Stat Med 2002 21 16 2313 24 10.1002/sim.1201 12210616

[B15] Mills E Wu P Ebert J Thorlund K Puhan MA Comparisons of High Dose and Combination Nicotine Replacement Therapy, Varenicline and Bupropion for Smoking Cessation: A Systematic Review and Multiple Treatment Meta-analysis Ann Med 2012 44 6 10 10.3109/07853890.2012.705016 22860882

[B16] Dias S Welton N Sutton A Ades A NICE DSU Technical Support Document 2 2011 A generalised linear modelling framework fro pairwise and network meta-analysis of randomised controlled trial 27466657

[B17] Thorlund K Imberger G Johnston B Walsh M Awad T Thabane L Evolution of heterogeneity (I^2) estimates and their 95% confidence intervals in large meta-analyses PLoS One 2012 7 7 10.1371/journal.pone.0039471 PMC3405079 22848355

[B18] Jackson D The implications of publication bias for meta-analysis’ other parameter Stat Med 2006 25 17 2911 21 10.1002/sim.2293 16345059

[B19] Rucker G Schwarzer G Carpenter JR Schumacher M Undue reliance on I(2) in assessing heterogeneity may mislead BMC Med Res Methodol 2008 8 79 10.1186/1471-2288-8-79 19036172 PMC2648991

[B20] Biggerstaff BJ Tweedie RL Incorporating variability in estimates of heterogeneity in the random effects model in meta-analysis Stat Med 1997 16 7 753 68 10.1002/(SICI)1097-0258(19970415)16:7<753::AID-SIM494>3.0.CO;2-G 9131763

[B21] DerSimonian R Laird N Meta-analysis in clinical trials Control Clin Trials 1986 7 3 177 88 10.1016/0197-2456(86)90046-2 3802833

[B22] Brockwell SE Gordon IR A comparison of statistical methods for meta-analysis Stat Med 2001 20 6 825 40 10.1002/sim.650 11252006

[B23] Hartung J Makambi K Reducing the Number of Unjustified Significant Results in Meta-analysis Comm Stat 2003 32 4 12

[B24] Spiegelhalter D Best N Carlin C van der Linde A Bayesian measures of model fit and complexity J Roy Stat Soc Ser B 2002 64 4 57

[B25] Lunn D Spiegelhalter D Thomas A Best N The BUGS project: Evolution, critique and future directions Stat Med 2009 28 25 3049 67 10.1002/sim.3680 19630097

[B26] The R Core T R: A Language and Environment for Statistical Computing 2005 Vienna, Austria: R Foundation for Statistical Computing

[B27] Awad T Brok J Thorlund K Hauser G Mabrouk M Stimac D Pegylated interferon versus non-pegylated interferon for chronic hepatitis C 2009 protocols: Cochrane database of systematic reviews 10.1002/14651858.CD005441.pub3 PMC11053364 24585509

[B28] Awad T Thorlund K Hauser G Stimac D Mabrouk M Gluud C Peginterferon alpha-2a is associated with higher sustained virological response than peginterferon alfa-2b in chronic hepatitis C: systematic review of randomized trials Hepatology 2010 51 4 1176 84 10.1002/hep.23504 20187106

